# Direct activation of STING in the tumor microenvironment with synthetic cyclic dinucleotide derivatives leads to potent and systemic tumor-specific immunity

**DOI:** 10.1186/2051-1426-2-S3-O10

**Published:** 2014-11-06

**Authors:** Leticia Corrales, Laura Hix Glickman, Sarah M McWhirter, David B Kanne, Kelsey E Sivick, Edward Lemmens, Justin J Leong, Ken Metchette, Thomas W Dubensky, Thomas Gajewski

**Affiliations:** 1 University of Chicago, Chicago, IL, USA; 2 Aduro Biotech, Inc., Berkeley, CA, USA

## 

Innate immune sensing in the tumor microenvironment is a critical step in promoting tumor infiltrating lymphocytes (TILs) and spontaneous anti-tumor T cell priming. Transcriptional profiling analysis of melanoma patients has revealed that tumors with a T cell-inflamed immunophenotype are characterized by a type I IFN (IFN) transcriptional signature. Studies in mice support the notion that IFN produced by tumor-resident dendritic cells (DCs) plays a critical role in spontaneous T cell priming against tumor antigens, which is dependent upon the host STING pathway. Preliminary data using the mouse STING agonist DMXAA injected intratumorally (IT) revealed potent tumor regression and induction of a robust anti-tumor T cell response. However, DMXAA does not engage human STING, necessitating alternative pharmacologic approaches to stimulate this pathway for clinical translation. We therefore synthesized a panel of cyclic dinucleotides (CDNs) that varied by purine nucleotide base, internucleotide phosphate bridge linkage, or by substitution of non-bridging oxygen atoms in the phosphate bridge with sulfur. We screened and selected from among these compounds based on their capacity to activate all known human STING alleles expressed in stably transformed reporter cell lines, stimulate the activation of human PBMCs, and impact significant antitumor efficacy in several mouse tumor models, without significant local or systemic toxicity. Strikingly, direct IT injection of particular CDN derivative molecules into two week-established flank B16 melanoma, CT26 colon, or 4T1 breast tumors profoundly inhibited tumor growth that was durable and correlated with induction of lasting systemic antigen-specific CD8^+ ^T cell immunity that conferred complete protection against tumor re-challenge, or significantly inhibited the growth of distal untreated tumors. Induction of cytokines, tumor antigen-specific immunity, and antitumor efficacy was entirely STING-dependent. We selected dithio-[Rp,Rp]-c[A(2',5')pA(3',5')p], a synthetic CDN molecule that has significantly higher activity than natural STING ligands, as the lead molecule for continued development. The synthetic CDN molecule described here was significantly more potent than IT TLR ligands, indicating its high translational potential as an approach to elicit effective unbiased T cell priming against an individual's unique tumor antigen repertoire.

